# Human motion capture for movement limitation analysis using an RGB-D camera in spondyloarthritis: a validation study

**DOI:** 10.1007/s11517-021-02406-x

**Published:** 2021-09-01

**Authors:** Manuel Trinidad-Fernández, Antonio Cuesta-Vargas, Peter Vaes, David Beckwée, Francisco-Ángel Moreno, Javier González-Jiménez, Antonio Fernández-Nebro, Sara Manrique-Arija, Inmaculada Ureña-Garnica, Manuel González-Sánchez

**Affiliations:** 1 Departamento de Fisioterapia, Instituto de Biomedicina de Málaga (IBIMA), Universidad de Málaga, Grupo de Clinimetría (F-14), Málaga, Spain; 2 Rehabilitation Sciences Research Department, Vrije Universiteit Brussel, Brussels, Belgium; 3 School of Clinical Sciences, Faculty of Health, Queensland University of Technology, Brisbane, QLD Australia; 4 Department of Rehabilitation Sciences and Physiotherapy, University of Antwerp, Antwerp, Belgium; 5 MAPIR-UMA Group, Department Ingeniería de Sistemas Y Automática, Instituto de Investigación Biomédico de Málaga (IBIMA), Universidad de Málaga, Málaga, Spain; 6 UGC de Reumatología, Instituto de Investigación Biomédica de Málaga (IBIMA) Hospital Regional Universitario de Málaga, Universidad de Málaga, Málaga, Spain

**Keywords:** Spondyloarthritis, Spinal mobility, Camera, BASFI, Motion capture

## Abstract

A human motion capture system using an RGB-D camera could be a good option to understand the trunk limitations in spondyloarthritis. The aim of this study is to validate a human motion capture system using an RGB-D camera to analyse trunk movement limitations in spondyloarthritis patients. Cross-sectional study was performed where spondyloarthritis patients were diagnosed with a rheumatologist. The RGB-D camera analysed the kinematics of each participant during seven functional tasks based on rheumatologic assessment. The OpenNI2 library collected the depth data, the NiTE2 middleware detected a virtual skeleton and the MRPT library recorded the trunk positions. The gold standard was registered using an inertial measurement unit. The outcome variables were angular displacement, angular velocity and lineal acceleration of the trunk. Criterion validity and the reliability were calculated. Seventeen subjects (54.35 (11.75) years) were measured. The Bending task obtained moderate results in validity (r = 0.55–0.62) and successful results in reliability (ICC = 0.80–0.88) and validity and reliability of angular kinematic results in Chair task were moderate and (r = 0.60–0.74, ICC = 0.61–0.72). The kinematic results in Timed Up and Go test were less consistent. The RGB-D camera was documented to be a reliable tool to assess the movement limitations in spondyloarthritis depending on the functional tasks: Bending task. Chair task needs further research and the TUG analysis was not validated.

**Graphical abstract:**

Comparation of both systems, required software for camera analysis, outcomes and final results of validity and reliability of each test.

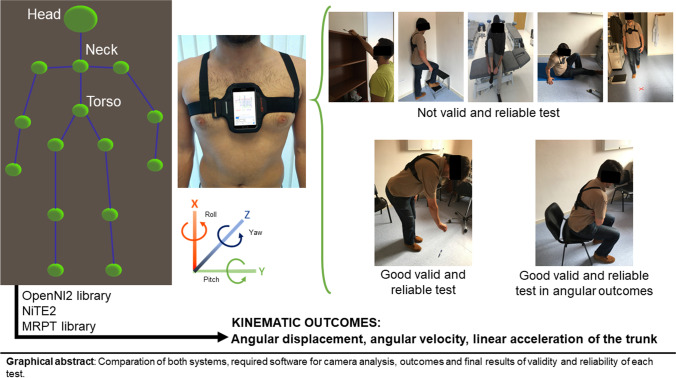

**Supplementary Information:**

The online version contains supplementary material available at 10.1007/s11517-021-02406-x.

## Introduction

Spondyloarthritis (SpA) is a chronic and rheumatic disease that affects the physical condition, work and quality of life of patients [1]. The prevalence is between 0.52 and 1.35% in North America, Europe and Asia [2]. A quick detection of SpA is a challenge due to the high prevalence of low back pain and the lack of knowledge about causality of this disease by general practitioners. Objective documentation is difficult due to the high frequency of absence of possible structural change in the sacroiliac joint [3, 4].

Owing to the negative impact on movement in SpA patients, analysing mobility and function is important and necessary in order to document the impact of disease [5]. Rheumatological questionnaires, for example the Bath Ankylosing Spondylitis Functional Index (BASFI), assess the physical limitations [6], but can be very influenced by environmental or psychological factors [7]. Functional tasks offer a more direct, objective and standardised observation of the active capacity of the subject than questionnaires [7]. Results of analysing kinematics of the back during a functional task in SpA can be an indicator of the degree of functional capacity and quality of life [8].

A human motion capture system with a RGB-D camera or depth camera has a relevant application in research and industry due to its easy use [9] and could be a reliable and valid tool that helps to understand the damage that SpA can induce in these patients. A RGB-D camera has been useful in other health issues, such as Parkinson’s disease or subacute and chronic low back pain [10, 11]. Furthermore, the RGB-D camera has been checked by Moreno et al. 2017 in Timed Up and Go (TUG) test, showing positive reliability of the camera (ICC = 0.81–0.84) and adequate correlation with an inertial measurement unit (IMU) in balance tests (*r* = 0.59–0.98) [12]. Both studies agree with the claim that it is easy to analyse the movement with only one camera plugged to a computer, thus eliminating the use of wired, wearable or more complex devices [12, 13]. There are other studies that analyse the mobility of the trunk in rheumatology diseases but they are based on motion capture system with reflective markers or the colocation of some inertial sensors [14, 15]. These methods need more time in the patient preparation, have a high costs and are difficult to use in the daily clinical practice [16, 17].

The aim of this study is to validate a motion capture camera using an RGB-D camera for the trunk movement limitation analysis in SpA patients, especially internal validity and reliability. It was used to register the functional tasks taken from the BASFI questionnaire and the TUG test in order to analyse the kinematic data. The hypothesis is that the RGB-D camera is a reliable and validated option and can lead to document and understand the spinal movement limitations in these patients.

## Material and methods

### Design and participants

This study is a longitudinal prospective study registered in ClinicalTrials.gov (NCT03293095). Volunteered patients were recruited from the Rheumatology Area of the Regional University Hospital in Málaga (Spain) and measured from March 2018 to May 2018.

Participants were between 18 and 75 years and fulfilled the Assessment of Spondyloarthritis International Society (ASAS) criteria [18]. They had a minimum score of 4 in the Bath Ankylosing Spondylitis Disease Activity Index (BASDAI) questionnaire. People with peripheral arthritis in lower limbs, participating in a study with an experimental treatment, have severe cardiovascular disease and have a lower limb arthroplasty in the last 6 months and pregnant women were excluded.

### Sample size calculation

A previous calculation of the sample size has been made in order to find out how many subjects the study needs with a significance level of 0.05 and a power of 80% using G.Power 3.1 software. The correlation was searched to optimise the calculation. A study reported a measurement of the correlation of a motion capture system and an inertial measurement unit in SpA subjects [14]. The chosen correlation was an acceptable correlation (r = 0.6) in the study. The calculation of the total size was 17 subjects (t critical = 2.13).

### Ethical approval

This study has the Ethical Approvement of the Coordinating Committee for the Ethics of Biomedical Research of Andalusia (N28092017). Furthermore, the study was in accordance with the guidelines for Good Clinical Practice from the International Conference on Harmonisation and the principles of Declaration of Helsinki. The protection of personal data in accordance with the Organic Law 15/1999 of December 13 on Protection of Personal Data was guaranteed. The subjects received an informative document about the study and an informed consent was signed by the participant and the researcher before the test started.

### Measurement instruments

#### RGB-D camera

**Fig. 1:**
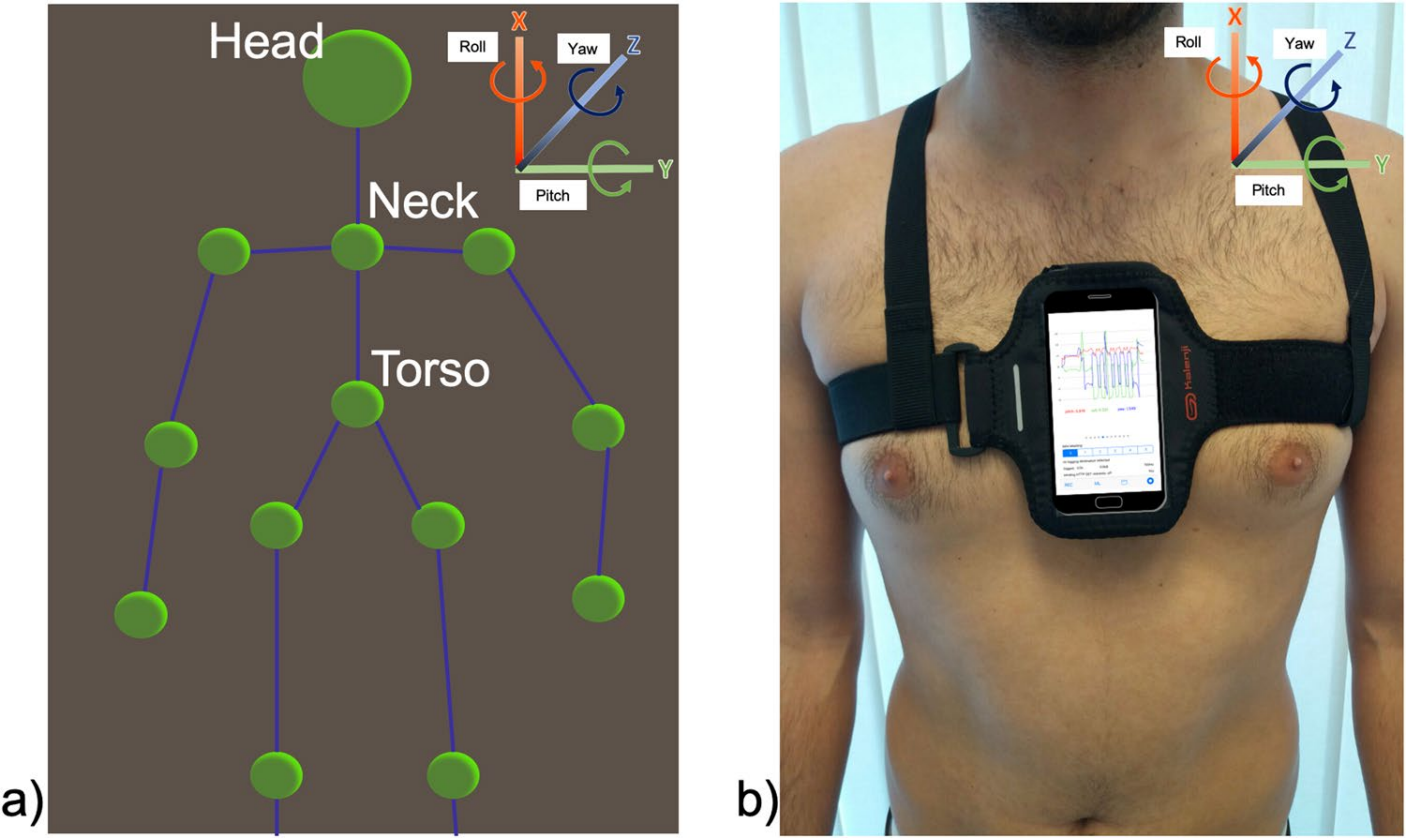
3D coordinate references system in both instruments. **a** 3D coordinate references and detected joints with the depth camera. **b** Inertial measurement unit placement and reference system.

An RGB-D camera by ASUS (Taipei, Taiwan) with these characteristics, working range: 0.8–3.5 m, size: 450 × 88 × 13 mm, depth image size: 60 fps, was used in this study. The distance between the camera and the participant was set to 2.5 m for the functional task and TUG test. The camera was placed at 45° with respect to the direction of the tested movements and at 90 cm of height. The sensor information collected by the RGB-D camera was used to construct a patient’s skeleton, composed of 3D coordinates of a set of 15 joints (Fig. 1a).

#### Inertial measurement unit from the smartphone

The IMU MP67B (InvenSense, San Jose, USA) from an iPhone6s (Apple Inc., Cupertino, USA) with iOS11 was used. The smartphone was placed on the medial third of the sternum and collected angular mobility along three axes [19]. The IMU showed high accuracy within medically acceptable limits (± 5°) [20].

The SensorLog® 2.2v app was used and processed the sensor data from the smartphone using the Core Location and Core Motion frameworks. The recording rate was set at 100 Hz. This app is available in the Apple App Store. All the data were saved in the smartphone memory and were sent to the computer for offline processing. A neoprene belt was used to the stabilise the smartphone on the chest (Fig. 1b).

### Functional tasks and TUG test

**Fig. 2:**
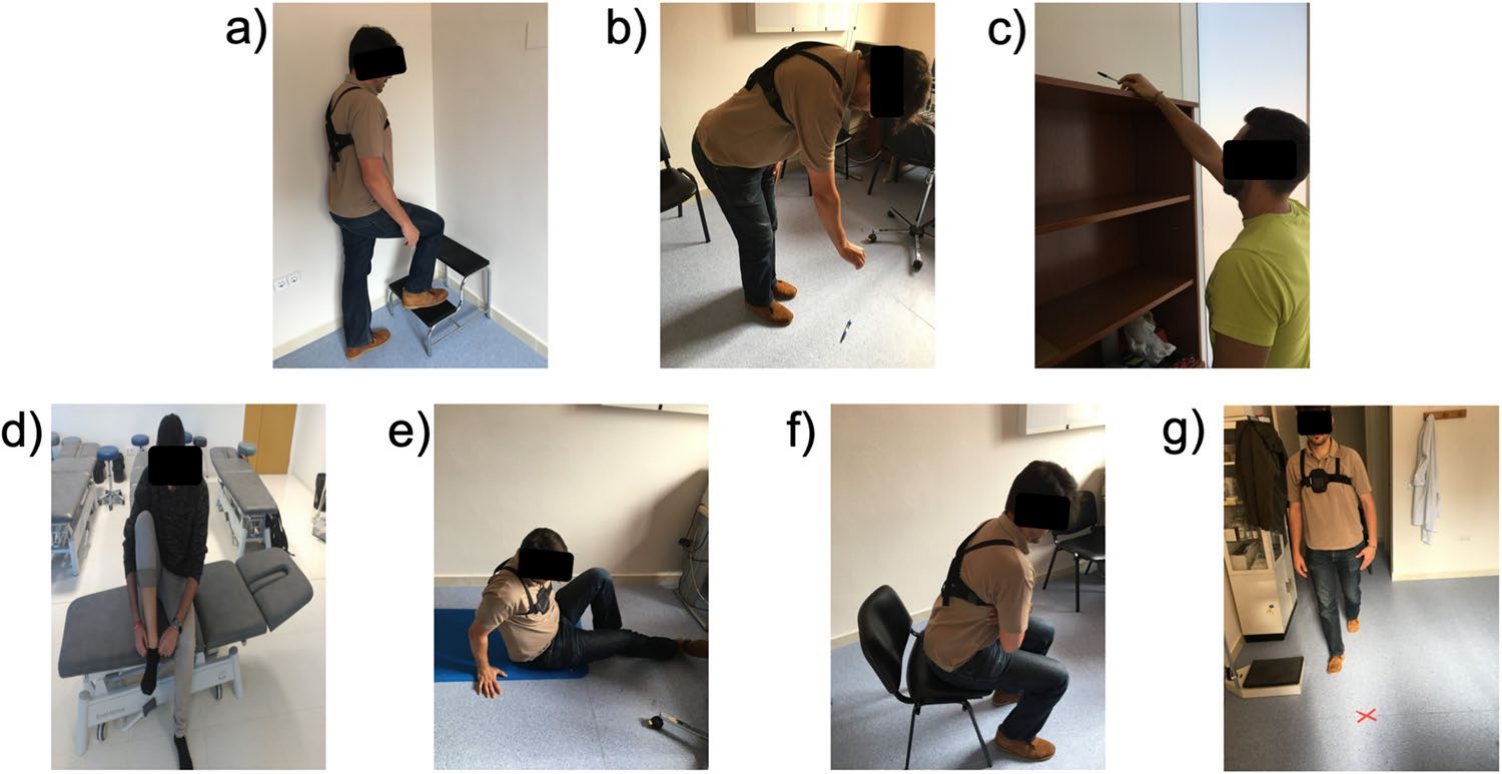
Functional tasks based on the BASFI items and Timed Up and Go test. **a** Climbing stairs. **b** Bending. **c** Reaching. **d** Putting socks. **e** Getting up from the floor. **f** Reclining and declining from a chair. **g** Timed Up and Go test

The kinematics was analysed with the motion capture system using functional tasks taken from the BASFI items previously carried out and justified by Van Weely et al. [21]. There is a predominance of movement in the sagittal plane in these tasks that corresponds to the flexo-extension of the trunk (Fig. 2).

Climbing stairs (Stairs): Subject had to climb 2-step stairs without aid by placing one foot on each step (height and depth of each step = 15 × 30 cm) [21].

Pick something up from the floor (Bending): A pen was placed on the floor in front of the subject. The subject was asked to bend forward from the hips and pick up the pen without aid [21].

Take something from an elevated place (Reaching): Subject facing a shelf placed at patient’s head height + 15%. Patient was instructed to take a pen on the shelf without help or aid [21].

Putting on sock (Sock): Subject had to put on his sock on the dominant foot sitting without help or aid. The sitting height of the table was 44 cm [21].

Getting up from the floor (Floor): A mat was used for the comfort of the patient. The patient started in lying supine position on the mat. He was instructed to stand up in one movement without help to come to a standing position in front of the mat [21].

Standing up from a chair (Chair): A chair with a 44-cm sitting height was used. The patient was instructed to stand up and sit down from the chair without using their hands or aid. The natural posture was accepted and no instructions about posture were given [21].

TUG was also performed after the functional tasks. The patient started the test seated on a chair (44-cm seating height) and was asked to get up and walk until reaching a cone at 3-m distance from the chair, turn around it, return to the chair and sit down again, walking as fast as possible without running but comfortable for the patient [22].

### Patient-reported outcome measures and anthropometric data

Important questionnaires in the daily clinical practice for SpA and validated in Spanish were used. Questionnaires were BASFI (ICC = 0.68) [23], BASDAI (α = 0.87) [24] and the Spondyloarthritis International Society Health Index (ASAS-HI) (ICC = 0.84) [25].

Anthropometric information, such as age, weight, height and body mass index (BMI), was also recorded.

### Procedure

Each measurement took 60 min to complete the questionnaires, to prepare the participant and to carry out the functional tasks.

After filling out the questionnaires, the smartphone was placed on the patient and the motion capture area of the RGB-D camera was shown. Each test was explained to the participant in order to clarify the correct execution of the tests. They had the possibility to familiarise with each test before the data collection. Participants carried out as many repetitions as possible during 30 s per functional task. Three trials of TUG were recorded. Patients rested during 120 s between each functional task to prevent fatigue. The subject was in a static position at the beginning and at the end of each measurement during 5 s in order to enable the data processing.

### Data processing

The data processing was conducted by an external and blinded researcher. The data set was synchronised with the timestamp and visually with the graphic 5 s before and after each test. The cycles were defined visually using the graphs based on the repetitive patterns and the peaks in the flexion–extension displacement.

The third repetition was chosen to be included in the analysis because the participant could improve the technique of the task execution in the previous repetition before fatigue and that scenario could influence the performance. The three first repetitions were chosen for the reliability analysis.

If due to the severity of complaints in certain patients, a patient could not complete the third repetition of a test; the third variable could be obtained with an average of the first two tasks.

#### Data analysis of the RGB-D camera

A representation of the patient’s skeleton is composed of a set of 15 joints: head, neck, torso, left and right shoulders, left and right arms, left and right hands, left and right hips, left and right knees and left and right feet. The information of the depth and the skeleton was obtained through the software libraries OpenNI2 and NiTE2 respectively. At last, based on the location of the skeletal joints during the tests, it is possible to calculate the range-based parameterisation of the patient’s movement. This parameterisation gave the inclination angles. The software was developed for a previous study [12] and has been publicly released as part of the open-source software library called MRPT [26, 27].

The 3D positions of the joints labelled Neck (N) and Torso (T) were used to calculate the angles between them for the angular outcomes. This coincides with the body motion at the T7 level and the movement of the centre of mass induced by a trunk flexion. For the camera orientation, let P_N_ = (X_N_,Y_N_,Z_N_) and P_T_ = (X_T_,Y_T_,Z_T_) be the 3D spatial coordinates of the Neck and Torso joints as measured, respectively. Therefore, the equivalent flexion–extension (α) or pitch angles can be computed as [12]:

$${\alpha }= {arctan}(\frac{{X}{N}-{X}{T}}{YN-YT})$$

Regarding the linear acceleration, the 3D position of the head was used for the analysis. A double derivative over time was performed with the position of the joint labelled Head, which had been previously smoothed through a Savitzky–Golay filter to reduce noise [28]. Thus, linear velocity is first computed by taking the median of the time intervals between the measurements in order to avoid large errors in case of gaps in the reading sequence:

$$\widehat{\Delta t}=median\left\{{\Delta t}_{i}^{i+1},i=0\dots N-1\right\}$$

Then, given the differences among the positions (∆p) of the head between time steps, the velocity is straightforwardly computed through:

$$V=\frac{\Delta p}{\widehat{\Delta t}}$$

Finally, the linear acceleration in each axis is determined by using again the median of the time intervals and the differences between the velocities (∆V) along time:

$$Acc=\frac{\Delta V}{\widehat{\Delta t}}$$

#### Data extraction of the inertial measurement unit

The inertial measurement unit was placed on the chest at T7 level. The smartphone’s orientation and the dimension of space were measured as follows: flexion–extension (α, pitch angle): rotation axis was Y, with positive data indicating flexion, and negative values indicating extension [12]. From the flexion–extension displacement, the other kinematic variables were calculated indirectly. The linear acceleration was obtained from the accelerometer in the Z axis.

### Outcome variables

The outcomes obtained directly were flexion–extension displacement (°) of the trunk, linear acceleration (m/s^2^) and time (s) for each subject. Antero-posterior angular velocity (°/s) was calculated as an indirect variable. Angular velocity was calculated indirectly based on the following formula: “velocity = displacement/time”. According to the coordinate reference in Fig. 1, the flexion–extension displacement was the pitch angle and the linear antero-posterior acceleration was the acceleration in Z. The number of repetitions was considered as an extra outcome variable. This information was extracted offline. An external and blinded researcher performed the data processing.

A set of so-called control points were used to mark different parts of the functional tasks. The variables mentioned before were computed for each interval. Every test had two control points: the starting position (A) and the ending position (B) of each test. Therefore, we measured the A → B interval. The data analysis changed in TUG due to complexity. In the case of TUG, the control points were as follows: starting point (A), stand up position (B), reaching the cone (C), the point immediately before the participant is starting to sit down (D) and the comeback to the starting point (E). Consequently, we measured the A → B, B → C, C → D and D → E intervals, respectively.

#### Statistical analysis

Mean and standard deviation (SD) were calculated for all the outcomes. All analyses were done with SPSS version 22 software (SPSS Inc., IL, USA).

##### Criterion validity

The criterion validity was measured by the correlation between the measurements of the motion capture RGB-D camera with the inertial measurement unit. Pearson’s correlation or non-parametric Spearman’s correlation test (r) was used according to the data distribution by the Kolmogorov–Smirnov test [29]. The correlation values were classified into three categories: poor (r ≤ 0.49), moderate (r = 0.50–0.74) and strong (r ≥ 0.75) [29].

##### Reliability

The reliability of the motion capture RGB-D camera was estimated by the interclass correlation coefficient two-way random-effects model 2.1 (ICC) and the standard error of measurement (SEM) comparing with the IMU. The reliability values were classified into four categories: poor (ICC ≤ 0.49), moderate (ICC = 0.50–0.74), good (ICC = 0.75–0.89) and excellent (ICC ≥ 0.90) [30].

## Results

Seventeen patients (n = 17) participated in the study and anthropometric and clinical data from subjects was calculated (Table 1). The mean age of the sample was 54.35 years (11.75) and the mean body mass index was 25.18 (3.36). The average scores of the BASFI, BASDAI and ASAS-HI questionnaires were 3.87 (2.05), 4.29 (2.21) and 6.23 (2.92), respectively.

**Table 1:** Anthropometric data and questionnaires scores in women and men

Variables	Women (n = 5)	Men (n = 12)	Total (n = 17)
Age (years)	54.6 (5.2)	54.2 (3.5)	54.3 (11.7)
Height (m)	1.6 (0.02)	1.7 (0.02)	1.6 (0.1)
Weight (kg)	59.3 (3.2)	76.0 (3.6)	71.1 (13.5)
BMI (kg/m^2^)	23.2 (1.2)	26.0 (0.9)	25.1 (3.3)
BASFI	3.8 (1.2)	3.8 (0.5)	3.8 (2.1)
BASDAI	3.7 (1.1)	4.5 (0.6)	4.2 (2.2)
ASAS-HI	5.4 (1.6)	6.5 (0.7)	6.2 (2.9)

*BASFI*, Bath Ankylosing Spondylitis Functional Index; *BASDAI*, Bath Ankylosing Spondylitis Disease Activity Index; *ASAS-HI*, Spondyloarthritis International Society Health Index.

The means (SD) of time, displacement, velocity and acceleration of each test according to the RGB-D camera and the IMU are reported in Table 2.

**Table 2:** Mean and standard deviation of the outcome variables of each test

Test	Repetitions	IMU time (s)	CAM time (s)	IMU angular displacement (°)	CAM angular displacement (°)	IMU angular velocity (°/s)	CAM angular velocity (°/s)	IMU lineal acceleration (m/s^2^)	CAM lineal acceleration (m/s^2^)
Stairs	4.6 (1.7)	2.9 (0.9)	2.9 (0.9)	16.6 (5.4)	12.9 (6.6)	5.7 (1.9)	4.4 (2.0)	0.7 (0.1)	1.0 (0.3)
Bending	10.7 (2.9)	1.6 (0.6)	1.6 (0.6)	42.6 (18.9)	55.2 (23.5)	27.2 (15.2)	34.8 (13.7)	0.6 (0.5)	0.8 (0.3)
Reaching	11.5 (4.0)	1.6 (0.8)	1.6 (0.8)	9.3 (4.1)	8.2 (5.3)	6.3 (3.7)	5.0 (2.7)	0.1 (0.1)	0.4 (0.2)
Sock	6.0 (1.7)	3.4 (1.5)	3.4 (1.5)	20.9 (14.2)	28.6 (16.2)	6.4 (3.8)	9.0 (4.6)	0.6 (0.3)	0.5 (0.3)
Floor*	2.5 (1.0)	5.1 (1.3)	5.1 (1.3)	99.9 (12.1)	75.4 (53.1)	20.4 (5.5)	16.8 (13.0)	2.1 (0.5)	2.5 (1.5)
Chair	8.0 (2.3)	2.1 (0.5)	2.1 (0.5)	31.8 (11.4)	36.8 (13.3)	15.7 (7.2)	17.4 (4.5)	1.3 (0.5)	1.0 (0.7)
TUG A-B		1.9 (0.3)	1.9 (0.3)	26.0 (12.3)	29.0 (12.3)	13.7 (4.4)	15.0 (5.9)	1.2 (0.3)	0.9 (0.3)
TUG B-C		1.6 (0.4)	1.6 (0.4)	8.2 (2.5)	18.3 (11.8)	5.8 (3.1)	12.8 (9.2)	0.5 (0.1)	0.9 (0.3)
TUG C-D		1.7 (0.6)	1.7 (0.6)	9.9 (2.5)	27.4 (12.8)	6.2 (2.7)	17.9 (10.8)	0.5 (0.1)	1.4 (0.4)
TUG D-E		2.3 (0.4)	2.2 (0.6)	30.8 (9.6)	39.0 (16.6)	13.5 (4.7)	17.1 (5.4)	0.9 (0.2)	1.6 (0.4)

*IMU*, inertial measurement unit; CAM, RGB-D camera; *TUG A-B*, TUG sitting to standing transition; *TUG B-C*, TUG from the chair to the cone; *TUG C-D*, TUG from the cone to the chair; *TUG D-E*, TUG standing to sitting transition.

*15 participants were included in this analysis because 2 participants could only perform 1 repetition due to their severity.

The validity and reliability outcomes of the RGB-D camera and the IMU are shown to be different in functional tasks (Table 3) and TUG test (Table 4). The time variable had the best correlation in all the tasks (r = 0.99–1.00). The Bending task obtained greater results of validity and reliability in all kinematic variables (r = 0.55–0.62, ICC = 0.80–0.88). The Chair task obtained good results in angular outcomes (r = 0.60–0.74, ICC = 0.61–0.72).

**Table 3:** Criterion validity and reliability obtained from the RGB-D camera and IMU in functional tasks

**Variables**		**r**	**SEM**		**ICC**	
			**CAM**	**IMU**	**CAM**	**IMU**
**Stairs**	*Time*	0.99	0.71	0.60	0.60	0.60
	*Angular displacement*	0.27	6.06	3.10	0.30	0.67
	*Angular velocity*	0.03	1.98	9.00	0.09	0.65
	*Linear acceleration*	0.11	0.20	0.11	0.66	0.63
**Bending**	*Time*	0.99	0.30	0.32	0.80	0.79
	*Angular displacement*	0.62	8.52	3.79	0.88	0.96
	*Angular velocity*	0.55	6.15	4.83	0.80	0.90
	*Linear acceleration*	0.60	0.16	0.09	0.81	0.97
**Reaching**	*Time*	0.99	0.42	0.38	0.80	0.79
	*Angular displacement*	− 0.15	3.43	1.72	0.60	0.83
	*Angular velocity*	− 0.14	1.82	1.96	0.55	0.72
	*Linear acceleration*	0.07	0.13	0.03	0.76	0.71
**Sock**	*Time*	0.99	0.89	0.88	0.67	0.67
	*Angular displacement*	0.67	8.70	5.34	0.79	0.86
	*Angular velocity*	0.59	3.41	2.26	0.46	0.66
	*Linear acceleration*	0.63	0.31	0.19	0.54	0.73
Floor*	*Time*	1.00	0.53	0.33	0.94	0.94
	*Angular displacement*	0.09	40.78	7.31	0.28	0.64
	*Angular velocity*	0.81	8.06	2.00	0.62	0.87
	*Linear acceleration*	0.19	1.41	0.34	− 0.01	0.60
**Chair**	*Time*	0.99	0.36	0.32	0.70	0.68
	*Angular displacement*	0.60	6.74	3.80	0.72	0.89
	*Angular velocity*	0.74	2.86	4.57	0.61	0.60
	*Linear acceleration*	0.37	0.20	0.32	0.90	0.65

*CAM*, RGB-D camera; *IMU*, inertial measurement unit; *TUG A-B*, TUG sitting to standing transition; *TUG B-C*, TUG from the chair to the cone; *TUG C-D*, TUG from the cone to the chair; *TUG D-E*, TUG standing to sitting transition.

*15 participants were included in this analysis because 2 participants could only perform 1 repetition due to their severity.

**Table 4:** Criterion validity and reliability obtained from the RGB-D camera and IMU in Timed Up and Go test

**Variables**		**r**	**SEM**		**ICC**	
			**CAM**	**IMU**	**CAM**	**IMU**
**TUG A-B**	*Time*	0.99	0.18	0.17	0.74	0.74
	*Angular displacement*	0.55	8.58	4.46	0.52	0.87
	*Angular velocity*	0.65	4.33	1.74	0.47	0.85
	*Linear acceleration*	0.01	0.18	0.18	0.59	0.65
**TUG B-C**	*Time*	0.99	0.21	0.25	0.78	0.75
	*Angular displacement*	0.58	7.71	1.74	0.32	0.54
	*Angular velocity*	0.90	6.98	2.03	0.43	0.57
	*Linear acceleration*	− 0.05	0.23	0.08	0.65	0.76
**TUG C-D**	*Time*	0.99	0.26	0.31	0.78	0.77
	*Angular displacement*	0.37	10.05	1.81	0.46	0.49
	*Angular velocity*	0.78	7.46	1.49	0.53	0.71
	*Linear acceleration*	0.14	0.39	0.06	0.36	0.85
**TUG D-E**	*Time*	0.98	0.35	0.24	0.75	0.74
	*Angular displacement*	0.35	10.71	3.75	0.52	0.85
	*Angular velocity*	0.61	5.06	2.09	0.14	0.81
	*Linear acceleration*	0.57	0.22	0.13	0.71	0.67

*CAM*, RGB-D camera; *IMU*, inertial measurement unit; *TUG A-B*, TUG sitting to standing transition; *TUG B-C*, TUG from the chair to the cone; *TUG C-D*, TUG from the cone to the chair; *TUG D-E*, TUG standing to sitting transition.

*15 participants were included in this analysis because 2 participants could only perform 1 repetition due to their severity.

## Discussion

The aim of this study was to present results of the validation and reliability of a motion capture system with a in SpA patients. The kinematic outcomes from the camera showed moderate to good results in validity and displacement in Bending and Chair tasks, but other functional task had poor validity (r < 0.50), poor reliability (ICC < 0.50) or both. Time obtained the strongest results, but displacement, velocity and acceleration were variable. According these results, the camera may not be a valid and useful method to analyse the functional tasks taken from BASFI, but it was found that specific tasks can be reliable and transferable to the daily clinical practice using this device, such as the Bending task and Chair task.

### Functional tasks

Time variable registered by the smartphone was the best correlated variable when compared with data from the RGB-D camera during the six functional tasks. There was an excellent correlation between the IMU and the motion capture system (r = 1.00–0.98) and a good reliability of the camera (ICC = 0.60–0.94) similar to the IMU (ICC = 0.60–0.94). Time results coincided with Moreno et al. [12] and they showed a strong validity and reliability of the depth camera when compared with a IMU in health people during balance tests (r = 0.76–0.97, ICC = 0.84–0.93).

Bending and Chair tasks had the best results in the analysed properties among other functional tasks. Other previous studies with low back pain patients showed similar results as this study in the displacement, velocity and acceleration outcomes (r = 0.53–0.80, ICC = 0.55–0.84) [11]. Bending task showed moderate to excellent results in all the outcomes with an RGB-D camera, but the validity to analyse the linear acceleration in Chair task was low (r = 0.37). In terms of displacement, this study improved the correlation obtained by a VICON system (r = 0.48) in a maximal trunk flexion, which can be compared with the Bending task [10]. Also in the same study, they validated with excellent results the Sit to Stand test, assessment test similar than Chair task (r = 0.99), in people with Parkinson’s disease [10]. This study is not comparable with our study because they decided to measure the linear movement of the head instead of the angular flexion of the trunk. Another study in ankylosing spondyloarthritis used a video-based motion capture system with markers and they obtained excellent results in contruct validity (r = 0.69–0.87) and reliability (ICC > 0.90, SEM = 0.37–5.32) [15]. Although there are a few results not favourable to the validity of the RGB-D camera, the discussion with other studies regarding these tests shows that the Bending and Chair tasks can be used to analyse the movement of the trunk in these patients.

The functional tasks showed a different correlation in the displacement: poor in Stairs (r = 0.27), Reach (r =  − 0.15) and Floor (r = 0.09); moderate in Sock (r = 0.67), Bending (r = 0.62) and Chair (r = 0.60). The tasks with better correlation were those where the flexo-extension trunk mobility was larger, more than 28° according to this study. This fact agreed with other validation studies where small or fine movements could be difficult to detect by the RGB-D camera system [10, 31, 32]. There is a wide displacement in the Floor task, but it is a task where the mobility around the three axes is very evident. This could cause errors in the data collection of the camera. These results agree with those shown in other study about the correlation and reliability in tasks with a large displacement of the trunk [11].

The reliability of the motion capture system was different between the tasks (Table 3). Regarding displacement, the tasks with better reliability regarding the camera, as in the correlation, the tasks with more mobility in a single axis (Bending ICC = 0.88, Sock ICC = 0.79, Chair ICC = 0.72). The IMU collected information with better reliability than RGB-D camera in these tasks (Bending ICC = 0.96, Sock ICC = 0.86, Chair ICC = 0.89). If we compare other functional tests such as Single Leg Squat or Drop Vertical Jump, these tests show similar results in the trunk flexion (ICC = 0.83–0.93) [31]. On the other hand, the reliability of accelerations showed better information as a whole than correlations (ICC = 0.54–0.90) except Floor task (ICC =  − 0.01). Despite this, the Floor task obtained good results in the angular velocity and acceleration (r = 0.81–0.85, ICC = 0.62–0.72). This information may be important in future studies since it is a difficult task for the patient with spondyloarthritis and can help to classify severity according to their function.

### Timed Up and Go Test

Regarding displacement, this study found a moderate correlation and reliability in the first interval of the test (r = 0.55, ICC = 0.52) and Moreno et al. [12] obtained better results in the first and last intervals (r = 0.67, ICC = 0.83). The working area of the camera and the total distance of the test could affect differently in each study due to the subject is at risk of getting too close to the camera when performing the intermediate intervals and there may be an exceptional loss of signal in the recording of the camera. The loss of signal may be a reason for the lesser strength in the acceleration results.

The reliability results show moderate results in the first and last intervals (ICC = 0.52). Vernon et al. [33] show better values in the trunk flexion (ICC = 0.73) and the velocity (ICC = 0.93) in people who suffered a stroke, as did Moreno et al. [12] (ICC = 0.83–0.84) [12]. An important limitation of this study is the positioning of the camera at the same distance of the turn point [33]. This positioning focuses on the analysis on the sit-to-stand and stand-to-sit phases, not on the whole test. Our study tried to collect the whole test but the working range of one camera may not be enough to achieve reliable information about the performance.

### Strengths and limitations of the study

The present study offers a series of strengths and weaknesses, for example patients who cannot perform the floor task because of the severity of his condition. It is a potential limit in order to take into account when analysing the results, but perhaps we did not limit enough the level of severity in order to find homogeneity and more accurate results. We decided to take the average of the first two repetitions to continue with the same structure analysis. The use of different reference systems to compare the kinematics may be another limitation to consider. The representation recorded by the camera is a virtual body created in the space without any physical marker or sensor. The sensor placed in contact with the participant does not obtain the same information because the references are different, and this can lead to a decrease in the correlation between them. Other studies compare the depth camera to a 3D Vicon system as gold standard [10, 34]. The reason of using an inertial measurement unit in this study is because the depth camera was successfully correlated in a previous study with a inertial sensor [12] and the chosen camera and smartphone references measured the movement of the centre of mass which is a relevant motion descriptor [35]. Returning to the information previously mentioned, similar data was obtained in this study and in others that used a 3D Vicon system [10, 34]. Another relevant point is related to the problems caused with the position and scope of the camera, and the overlapping joint. We consider for future studies the use of more than one camera to obtain complete and valid information [13, 36] but several crosstalk issues have to be solved. Two interesting points should be taken into account to overcome these results: an improvement in the procedure and the collection of the RGB-D camera system, and finding new clinical outcomes different from trunk movement that are easier to detect by the camera and are more clinically relevant [37].

On the contrary, this is the first kinematic study in SpA using an easy-to-use motion capture system, cheaper than other diagnostic imaging tests and that minimises the subjectivity of the evaluations of functional limitations by questionnaires. The ability to capture the patient’s movements automatically without inertial sensors or reflective marks helps to spend less time assessing the subject and correct errors derived from the different criteria of the evaluators [38]. Therefore, this is the starting point to determine which tests can be crucial for the assessment of these patients and it can present a future basis for further studies where reference values and ambulatory indexes can be obtained to distinguish patients from healthy subjects and different degrees of severity.

### Conclusion

The human motion capture RGB-D camera could be a reliable tool to assess the movement limitations in SpA depending on the functional task: Bending task. Further research for the Chair task in this clinical population is necessary. The registration of the TUG and other tasks is shown to be less reliable. In addition, the camera can be a useful tool to measure the time during the task without the disadvantages of human contact. This result can start to lead the way for a better evaluation of the physical limitations of SpA patients through more objective and direct assessments.

## Supplementary Information

Below is the link to the electronic supplementary material.

Supplementary file1 (XLSX 37 KB)
